# The role of occludin in vascular barrier function in vivo: do we need to re-examine?

**DOI:** 10.1186/s13054-020-03178-w

**Published:** 2020-07-22

**Authors:** Ruyuan Zhang, Yiyun Liu, Dechang Chen, Yaoqing Tang

**Affiliations:** Department of Critical Care Medicine, Rui Jin Hospital, Shanghai Jiao Tong University School of Medicine, Shanghai, 200025 China

Dear editor,

Septic patients often undergo altered mental status. One possible reason for this brain dysfunction is thought to be related to vascular leakage caused by systemic inflammation. The recent article by Erikson et al. [1] showed that major tight junctions protein occludin in brain autopsy specimens in sepsis was downregulated and that the downregulation of occludin was related with severe organ dysfunction and systemic inflammation. While these findings are novel and objective, we feel it may be necessary to interpret the data with caution regarding to equating the decrease of occludin expression with blood-brain barrier dysfunction in this and other related research.

Since its discovery by Shoichiro Tsukita, a pioneer in the field of tight junction, most of the data in literature about the causal role of occludin in barrier function are from in vitro experiments. However, occludin-deficient mice do not display a perturbation of barrier function, with a complex pathophysiological and inexplicable phenotype [2] which even disappointed its discoverer Shoichiro Tsukita. Moreover, few reports regarding the in vivo barrier function of occludin mainly focused on epithelial cells [3]. Surprisingly, research from Charles M. Rice’s lab showed that occludin acts as a hepatitis C virus entry factor [4] and thus won the 2016 Albert Lasker Award. In another recent report dedicated to the memory of Dr. Shoichiro Tsukita, occludin deficiency in mice causes deafness while not affecting the tight junction structure or barrier [5].

Therefore, due to the lack of vascular barrier disruption in occludin-deficient mice, additional in vivo animal experiments may be needed to make the conclusions for those studying the role of occludin in vascular barrier function in vivo and those using occludin expression as an indicator of blood-brain barrier function more reliable. However, there is a possibility that occludin^−/−^ mice may be able to compensate for occludin loss. It also cannot be excluded that the level of occludin plays limited roles under normal physiological conditions but modulates the sensitivity of blood vessels to pathophysiologically relevant stressors. Some questions that need to be answered in the future include the following: Will postnatal endothelial-specific occludin knockdown in mouse brain lead to vascular leak? Are occludin-deficient mice more sensitive to sepsis? Will endothelial-specific occludin overexpression prevent or limit sepsis-induced brain vascular leak in vivo?

## Data Availability

Not applicable.
